# Changes in the pattern of suicides and suicide attempt admissions in relation to the COVID-19 pandemic

**DOI:** 10.1007/s00406-022-01448-y

**Published:** 2022-07-04

**Authors:** Christine Reif-Leonhard, Dorothea Lemke, Franziska Holz, Kira F. Ahrens, Christoph Fehr, Markus Steffens, Michael Grube, Christine M. Freitag, Sarah C. Kölzer, Sabine Schlitt, Rebekka Gebhardt, Theresa Gädeke, Helga Schmidt, Ferdinand M. Gerlach, Kira Wolff, Michael Stäblein, Nora Hauschild, Inga Beig, Louisa Wagner, Juliane Müller, Marcel A. Verhoff, Christiane Schlang, Andreas Reif

**Affiliations:** 1 Department of Psychiatry, Psychosomatic Medicine and Psychotherapy, University Hospital Frankfurt-Goethe University Frankfurt, Heinrich-Hoffmann-Str. 10, 60528 Frankfurt am Main, Germany; 2 Institute of General Practice, Goethe University Frankfurt, Frankfurt am Main, Germany; 3 Institute of Legal Medicine, University Hospital Frankfurt-Goethe University Frankfurt, Frankfurt am Main, Germany; 4 Vitos Klinik für Psychiatrie und Psychotherapie Hadamar, Hadamar, Germany; 5 Klinik Hohe Mark, Frankfurt am Main, Germany; 6 Catholic University of Applied Sciences Mainz, Mainz, Germany; 7 Department of Psychiatry, Städtisches Klinikum Frankfurt-Hoechst, Frankfurt am Main, Germany; 8 Department of Child and Adolescent Psychiatry, Psychosomatic Medicine and Psychotherapy, University Hospital Frankfurt-Goethe University Frankfurt, Frankfurt am Main, Germany; 9 Gesundheitsamt Frankfurt Am Main, Frankfurt am Main, Germany; 10 Department of Psychiatry, Agaplesion Markus Krankenhaus, Frankfurt am Main, Germany

**Keywords:** Suicide attempt, Completed suicide, COVID-19, Pandemic, SARS-CoV2

## Abstract

The consequences of the current COVID-19 pandemic for mental health remain unclear, especially regarding the effects on suicidal behaviors. To assess changes in the pattern of suicide attempt (SA) admissions and completed suicides (CS) in association with the COVID-19 pandemic. As part of a longitudinal study, SA admissions and CS are systematically documented and analyzed in all psychiatric hospitals in Frankfurt/Main (765.000 inhabitants). Number, sociodemographic factors, diagnoses and methods of SA and CS were compared between the periods of March–December 2019 and March–December 2020. The number of CS did not change, while the number of SA significantly decreased. Age, sex, occupational status, and psychiatric diagnoses did not change in SA, whereas the percentage of patients living alone while attempting suicide increased. The rate and number of intoxications as a SA method increased and more people attempted suicide in their own home, which was not observed in CS. Such a shift from public places to home is supported by the weekday of SA, as the rate of SA on weekends was significantly lower during the pandemic, likely because of lockdown measures. Only admissions to psychiatric hospitals were recorded, but not to other institutions. As it seems unlikely that the number of SA decreased while the number of CS remained unchanged, it is conceivable that the number of unreported SA cases increased during the pandemic. Our data suggest that a higher number of SA remained unnoticed during the pandemic because of their location and the use of methods associated with lower lethality.

**Supplementary Information:**

The online version contains supplementary material available at 10.1007/s00406-022-01448-y.

## Introduction

The Coronavirus disease 2019 (COVID-19) pandemic can be considered as a world-wide macro-stressor, which has, and continues to, heavily impact people’s life regarding their freedom, health, and socioeconomic situation. Several factors, such as severe acute respiratory syndrome coronavirus 2 (SARS-CoV2) exposure itself, as well as governmental responses to the pandemic, must be considered when interpreting the consequences of the pandemic. Thus, both infection per se, as well as lockdowns [1] and other consequences of the pandemic, might contribute to environmental risk for mental disorders, especially in vulnerable populations [2]. Unsurprisingly, it has been speculated that the pandemic in general, and quarantine measures in particular, will have a major ongoing impact on public mental health [3].

That said, the effect of the pandemic on general mental health is still under debate, but the available evidence suggests that there have been several negative consequences. For instance, in a vulnerable group of previously healthy individuals, we revealed a deterioration in their mental state during the pandemic [4, 5]. In line with this, studies on mental health in children and adolescents [6, 7], as well as adults from all over the world [8, 9], point to a significant and relevant 25% (adults) to 50% (children, adolescents) increase in depressive and anxiety symptoms in the general population. While these studies mainly rely on symptom checklists rather than established diagnoses, the data are nonetheless worrisome with respect to overall mental health in all age groups and demographics.

Furthermore, patients already suffering from mental disorders are likely to have disproportionately suffered from the pandemic’s consequences for a variety of reasons: financial hardship, loneliness and loss of social networks, reduced access to care, to name but a few. Thus, deterioration of their mental health status has been feared—with a concomitant increase in suicide rates. Indeed, model calculations argued that an increase in unemployment rates will lead to excess mortality due to CS [10]. In addition to such socioeconomic consequences, direct neurobiological effects of SARS-CoV2 infection on the brain that may result in increased suicidal behavior have been hypothesized [11]. Taken together, these consideration led to the concern that that the COVID-19 pandemic would lead to an increase in suicidal behaviors [12].

However, a recent meta-analysis of studies from across 21 countries, which included almost 20,000 suicide cases, did not support this hypothesis. Rather, this analysis showed that suicide rates from April 2020 to July 2020 remained statistically unchanged from pre-pandemic levels [13]. Data on suicide attempts, however, are sparse, particularly as public epidemiological and administrative databases often provide insufficient details on critical variables of suicide attempts: underlying mental disorders, methods and means, as well as sociodemographic data, are typically incompletely assessed in routine settings. Cross-sectional assessments are also suboptimal, as secular trends or other confounding variables are not properly accounted for using such approaches. On the other hand, longitudinal data collected from the same catchment area using standardized approaches can overcome such limitations and, thus, provide meaningful information about a possible change in these variables. Often, such data are only available in the context of long-term research projects. These arguments were central to the initiation of the project “FraPPE” (*Frankfurt project on suicide prevention using evidence-based measures*) in 2017, which comprises community-based suicide prevention measures in the city of Frankfurt am Main (765.000 inhabitants in 2020). One part of this project is the systematic assessment of suicide attempts as well as completed suicides in Frankfurt by installing a reporting system in all four psychiatric hospitals that participate in communal healthcare. To achieve this, all patients who are admitted for a suicide attempt (SA) have been documented since 2018. Additionally, completed suicides (CS) were systematically evaluated through the Institute of Forensic Medicine and compared with data from the Communal Health Authority. Therefore, our study provides the unique opportunity to compare pre-pandemic “baseline” data with data during the pandemic regarding both suicide attempts and completed suicides in a major metropolitan area.

## Methods

### Overall study design

The present analysis is part of the suicide prevention program “FraPPE” (Frankfurt project to prevent suicides by evidence-based measures), funded by the German Health Ministry from 2017 to 2021 (Grant no. ZMVI1-2517FSB136). The program used a multi-level communal intervention in the city of Frankfurt am Main (inhabitants in 2020: ca. 765.000; the number of inhabitants did not meaningfully change from 2019 to 2020). All psychiatric hospitals in Frankfurt/Main participated in the project, as well as the communal health authority, and the Institutes of General Practice and Legal Medicine of the Goethe-University Frankfurt. Interventions comprised establishment of a 24/7 suicide prevention hotline, implementation of a specialized psychotherapy program focusing on prevention of recurrence after SA (ASSIP), education and training of GPs, communal outreach to emergency services to increase the rate of referrals to mental health services after SA and strengthening the communal suicide prevention network. The project started in 2017 and the measures ran until December 2020. Method monitoring and geo-analyses were important further parts of the project. The study will be described in detail in a later publication (in preparation); further information can be retrieved from the website frappe-frankfurt.de. Part of the project was as systematic assessment of routine data on suicide attempts and completed suicides. For the latter, forensic pathologists were called to the sites where completed suicides took place. The data collected on site and at the Institute of Legal Medicine were, on one hand, compared with the police investigation results and supplemented where necessary. On the other hand, the completed suicide cases were compared with the data from death certificates collected at the Communal Health Authority. In doing so, we could obtain the most complete data on completed suicide in Frankfurt/Main. To record suicide attempts, a structured documentation was implemented at all four psychiatric hospitals that provide inpatient mental health services in Frankfurt am Main (University Hospital Frankfurt, Agaplesion Markus Krankenhaus, Städtisches Klinikum Hoechst, Klinik Hohe Mark). Routine demographic and medical information of every in- and outpatient presenting to one of the hospitals after a suicide attempt was collected and entered into a case report form (CRF). Completeness of measurements was supported by integrating this assessment in the clinical SOPs, regular supervision in clinical routine and manual comparison of the clinical information system with the CRFs. The authors assert that all procedures contributing to this work comply with the ethical standards of the relevant national and institutional committees on human experimentation and with the Helsinki Declaration of 1975, as revised in 2008. As only routine data were collected, no Ethical Approval and Consent is required according to the Medical Association's professional code of conduct.

### Suicide attempts: assessed variables

Suicide attempt (SA) was defined as deliberate self-harm with intend to die, irrespective of fatality probability [14]. Deliberate self-harm without intend to die, i.e., accidental drug overdose or self-harm for emotional tension relief without suicidal ideation e.g., in the context of borderline personality disorder, were not considered as suicide attempts. This was operationalized by development of a 24-item questionnaire to record SA in the psychiatric hospitals. The questionnaire and a study folder including all necessary information were presented and explained as part of in-house training courses in all participating hospitals. Immediately after admission, the clinician who treated and diagnosed the patient, filled out the case report form (CRF) and forwarded it to the responsible on-site FraPPE-team member (RG, TG, HS or LW). After quality checking, the anonymized CRFs were sent to the Department of Psychiatry of the University Hospital Frankfurt, where one of the authors (CS) centrally controlled every CRF regarding completeness and quality of the data and, if necessary, approached the responsible clinician in case of problems. Additionally, one percent of all CRFs which were drawn randomly from the overall sample were quality checked by the Institute of General Practice. Socioeconomic, individual and clinical variables were extracted from routine data in analogy to the European MONSUE project [15]. In the CRF, sociodemographic information, time and place of suicide attempt, main underlying psychiatric diagnosis, possible proximal triggers of SA, method of SA, consequences of SA (especially further medical and psychiatric treatments) and legal basis for admission were recorded. Completion of the CRF within 48 h upon admission was strived for; the CRF was completed by the clinician who saw the patient upon admission. Data are presented for all hospitals combined.

### Completed suicides: assessed variables

Completed suicide (CS) was defined as intentional self-harm with fatal outcome [14]. All CS cases from the Frankfurt metropolitan area were included, regardless of whether the person died immediately at the suicide scene or was still hospitalized and died in the course of the consequences of the intentional self-harm. Intentional self-harm was diagnosed in collaboration with the criminal investigators and the forensic pathologist, who were called to the suicide scene, and was primarily determined by existing suicide notes and interviews with family members, friends, or acquaintances, as well as medical records found. Deaths that could not be clearly attributed to intentional self-harm were excluded. A CRF was completed. When possible, the corpse was autopsied at the Institute of Legal Medicine: either on the order of a judicial postmortem examination by a local court, or, if no order was issued, with the consent of the relatives. In all autopsied cases, additional chemical-toxicological examinations were performed to detect medications, alcohol, and drugs in various body fluids and tissue samples. The number of CS was compared and cross-checked between the Municipal Health Authority and the Institutes of Legal Medicine and General Practice.

### Statistical analyses

To examine the impact of the first COVID-19-related lockdown in 2020, we compared the data from March 2019–December 2019 (hereafter termed “baseline”) to the data from March 2020–December 2020 (hereafter termed “pandemic”). An overview on lockdown measures, beginning in March 2020, is given here [5]. All outcomes are presented using descriptive statistics; for continuous, normally distributed variables, mean, median and for binary and categorical variables frequency and percentages will be provided. For statistical testing, a significance level of alpha = 0.05 (5%) and two-sided hypothesis testing (if not specified otherwise) are applied. If variables are not normally distributed, non-parametric tests are conducted.

For data management, statistical analysis, and graphical visualization R version 3.6.1 (R Core Team, 2019) and RStudio version 1.2.5 (RStudio Team, 2020) were used.

## Results

### Absolute changes in the number of suicide attempts (SA) and completed suicides (CS)

During baseline conditions (March–December 2019), 430 SA were documented (thereof 210 females, 49%). In contrast, within the pandemic, i.e., March–December 2020, 296 SA were recorded (thereof 145 female, 49%). The incidence rate (total number of SA per inhabitants) differs significantly between baseline and the pandemic with a decrease of almost 30% observed during the pandemic (Incidence rate ratio (IRR) = 0.69, *p* < 0.0001). Regarding CS cases, 86 were recorded during baseline (thereof 32 females, 37%). During the pandemic (March–December 2020), 81 CS cases were documented (thereof 22 females, 27%). Thus, in contrast to SA, the incidence rate of CS did not differ significantly between baseline and the pandemic (IRR = 0.94, *p* > 0.05).

### Age and sex distribution of SA and CS

Table 1 provides an overview on the age and sex distribution during baseline and the pandemic. For SA, neither sex distribution nor average age was significantly different between baseline and the pandemic. From the 430 SA cases in 2019, 37 patients (8.6%) were under 18 years of age, while in 2020, 18 patients out of 296 (6.1%) were under 18 years old. The change in the percentage of minors was not significant (Pearson’s Chi-squared test, Chi^2^ = 1.6, *p* > 0.05, OR = 0.69). The mean age of CS was not significantly different (54.8 years before the pandemic vs. 53.1 years during the pandemic). Two persons under 18 years of age died from suicide in either period. A detailed overview on the age distribution is given in Supplementary Figs. 1 and 2.

**Table 1:** Age and sex distribution in suicide attempts and completed suicides from March–December 2019 and March–December 2020

	Suicide attempts			Completed suicide		
	03/19–12/19	03/20–12/20	*p* value (overall)	03/19 – 12/19	03/20–12/20	*p* value (overall
% female	49.3	45.6	0.292	37.6	27.2	0.202
Mean age (years)	38.8	41.9	0.065	54.8	53.1	0.530
Median age (years)	36	39		54.5	53.0	
Youngest (year)	11	13		16	16	
Oldest (year)	92	90		96	89	

### Sociodemographic factors in patients with suicidal behavior

All following data relate to adults only. Regarding the marital status, there were no significant changes between both periods: 46 vs. 47% of patients were single, 7 vs. 8% were divorced, 5 vs. 5% were widowed, 21 vs. 19% were married, 6 vs. 6% were cohabitating in 2019 vs. 2020, respectively (remainder to 100%: unknown, other, or not documented; 15 vs. 14%). A significant difference was noted in the composition of the household (Pearson’s Chi-squared test, Chi^2^ = 23.9, *p* = 0.013; Supplementary Table 1) purporting to an increased percentage of patients attempting suicide living alone. Moreover, the occupational status at baseline and during the pandemic was significantly different (Pearson’s Chi-squared test, Chi^2^ = 27.0, *p* = 0.0003; Supplementary Table 2). However, the values are hard to interpret since the most pronounced change occurred in the category “other”. There were no significant changes in citizenship. While in 2019, 95 patients attempting suicide had their principal residence outside of Frankfurt (24%), this number declined to 53 in 2020 (19%).

Regarding CS, no significant differences were found regarding household composition and occupations status, however, the power of this sample was likely too low to detect changes. The incidence rate of CS in individuals with non-German citizenship increased from baseline to the pandemic from 4.4/100.000 inhabitants to 8.8/100.000 inhabitants (IRR = 1.99, *p* > 0.05), especially in the group of non-German EU citizens (1.0/100.000 to 13.2/100.000) (IRR = 13.3, *p* < 0.001), while the rate for German citizens decreased from 14.3/100.000 to 11.5/100.000 (IRR = 0.8, *p* > 0.05).

### Diagnoses underlying suicidal behavior

There were no significant changes in the main psychiatric diagnoses underlying SA between baseline and the pandemic in adult patients (Supplementary Table 3). In completed suicide, the rate of individuals where no psychiatric diagnosis could be established was comparatively high; no significant changes in the distribution of diagnoses were found.

### Change of methods in SA and CS

With respect to the methods used in SA, we found a highly significant effect that the rate of intoxication-related SA significantly increased in adult patients during the pandemic (Pearson’s Chi-squared test, Chi^2^ = 50.1, *p* < 0.0001; Table 2). In CS, there was slight, but non-significant, decrease in fatal suicidal intoxications, which was mirrored by a numeric increase in falls from height and collisions with a railway, as well as drowning and sharp violence.

**Table 2:** Methods used in suicide attempts before and during the pandemic

Method according to ICD-10 X code (%)	Suicide attempts			Completed suicides		
	03/19–12/19	03/20–12/20	*p* value (overall)	03/19–12/19	03/20–12/20	*p* value (overall)
X60–X69 (intoxication)	50.6	77.3	< 0.001	25.6	17.3	0.084
X70 (strangulation)	7.4	2.2		29.1	18.5	
X80 (leaping from heights)	9.7	4.7		23.3	29.6	
X81–X82 (railway, traffic)	10.7	4.7		7.0	11.1	
XX71_79_83_84 (other injuries)	21.6	11.2		15.1	23.2	

### Time, date and location of SA and CS

**Fig. 1:**
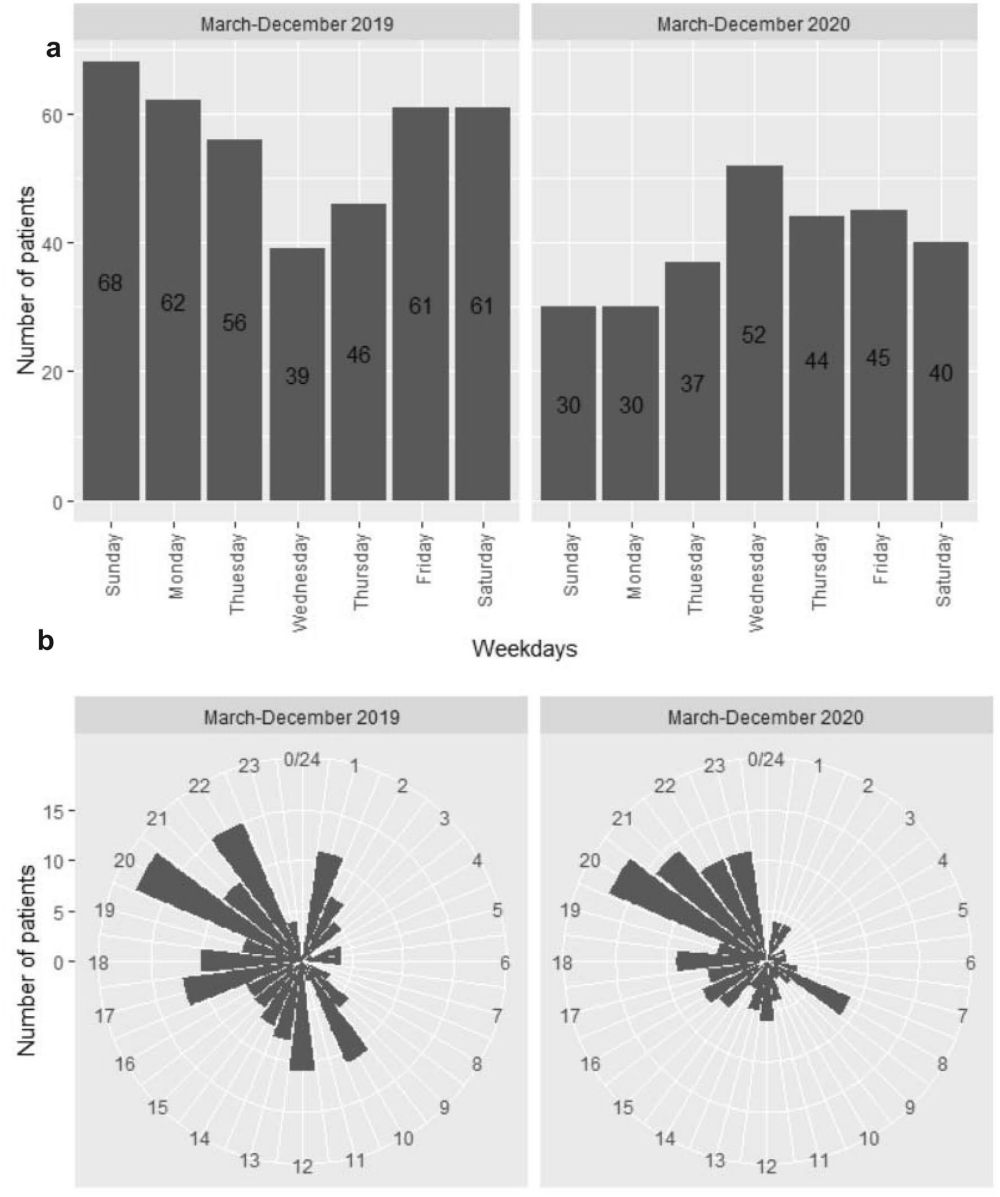
**a** Weekday distribution of suicide attempts at baseline and during the pandemic; **b** diurnal variation of suicide attempts at baseline and during the pandemic. Missing data *n* = 362 in total

Regarding the day of the week when a SA occurred, we observed a reversed pattern between the two datasets: while at baseline, the trough in SA was during the middle of the week, whereas Wednesday was the peak SA day during the pandemic (Pearson’s Chi-squared test, Chi^2^ = 19.3, *p* = 0.004; Fig. 1a). No significant changes were observed with respect to the hour of SA (Pearson’s Chi-squared test, Chi^2^ = 31.1, *p* > 0.05; Fig. 1b); most suicides during both periods occurred between 20.00 and midnight, although during the pandemic, almost no SA were documented between midnight and 8.00. No significant change was observed for the month of SA (not shown). No reliable data are available for time and weekday of completed suicides. The seasonal pattern of CS is shown in Supplementary Fig. 3.

A significant change was found in the location of SAs, as significantly less people attempted suicide elsewhere vs. at home during the pandemic (49 vs. 59%; Pearson’s Chi-squared test, Chi^2^ = 6.6, *p* = 0.01, OR = 0.69). If SA was attempted outside home, there was also a shift in the SA location, in that less suicides occurred in train stations or railways (Pearson’s Chi-squared test, Chi^2^ = 30.9, *p* = 0.02; Table 3). The number of CS committed at the registered address decreased from 69.8 to 53.1%, while the rate of CS at transport routes (railway, road) increased from 10.5 to 17.3%, as did CS in rivers/ponds (3.5–7.4%; all *p* > 0.05).

**Table 3:** Location of suicide attempts before and during the pandemic

Location (%)	03/19–12/19	03/20–12/20	*p* value (overall)
At home/missing data	48.6	58.6	< 0.001
Other city	1.3	0.0	
Other flat/hotel	2.6	2.9	
Refugee camp	0.3	0.0	
Other country	0.8	0.0	
Construction sites	0.3	0.0	
Bridges or river banks	4.3	3.6	
Drug healthcare system	0.3	0.0	
Airport	2.6	0.0	
Train station, Railways	9.9	4.3	
Hospitals	4.3	0.7	
Forests, greenspace	1.3	0.7	
Nursery homes	0.3	0.0	
Police stations	0.5	0.0	
Psychiatric hospitals	1.3	0.0	
Traffic	3.1	3.6	
Elsewhere outside	4.1	1.4	

Remainder to 100% is missing data/unknown

## Discussion

The effect of the COVID-19 pandemic, and the corresponding lockdown, on mental health is a matter of ongoing debate. Shortly after the first lockdown, it was speculated that the incidence of a number of mental disorders, but especially depression and anxiety, would increase as a direct consequence (e.g. [3]). The corresponding data that has been garnered to assess this hypothesis has been predominantly obtained from cross-sectional studies, which often employ screening measures without clinically relevant diagnostic assessment. Thus, the effect of the COVID-19 pandemic on mental health at the population level has not yet been firmly established. Suicide (CS) and suicide attempts (SA) can be considered as proxies for severe mental disease burden, given they are amongst the worst (and most extereme) outcomes of mental disorders. While a large, multi-country meta-analysis provided compelling evidence that the rate of CS did not significantly change in 2020 from pre-pandemic levels [13], there is surprisingly little data on SA. This might be because SA are often not documented systematically. In most countries, there are no SA surveillance systems, and even if SA are systematically assessed, there is limited data on other demographic or medical factors in this population. The FraPPE study, which ran between 2017 and 2021, is a communal suicide prevention project where one of the modules is systematic assessment of SA in all psychiatric hospitals in Frankfurt am Main. The number of unreported SA cases is likely to be significant given that only those cases that are admitted to psychiatric hospitals are recorded and as the CS/SA ratio in our sample was 1:5. However, a ratio of 1:10 for CS:SA has been calculated using data obtained in the nearby city of Würzburg in the MONSUE project [16]; a 1:20 ratio is assumed by the WHO [17]. Thus, these values can be used as a proxy to extrapolated the obtained datasets.

Our project reported 430 patient admissions to the four psychiatric hospitals. Thus, using the 1:10 ratio and a base rate of ca 90 suicides in Frankfurt p.a., we estimate to have seen approximately 50% of SA via our reporting system. Therefore, we believe that the FraPPE represents one of the most complete SA demographic databases available and the continuous assessment within a large catchment area enables the evaluation of secular trends in numerous SA variables. Moreover, it is important to emphasize that an important aspect of FraPPE was to raise awareness and to refer patients after SA to psychiatric care for follow-up evaluation. Thus, a major target group of this campaign has been emergency room staff and emergency medical services. Based on this, an increased referral rate can be assumed suggesting that our data on the absolute number of SA is leaning more towards the conservative side. To interpret our data, it is also important to consider potential confounders with respect to mental healthcare provision in the region. The four participating institutions (University Hospital, Agaplesion Markus Krankenhaus, Klinikum Hohe Mark and Städtisches Hospital Frankfurt-Hoechst) provide all inpatient services for the city of Frankfurt am Main and are obliged to admit all patients in the case of medical need. The overall number of inpatient psychiatric beds did not significantly change in these hospitals during the pandemic; therefore, variables regarding psychiatric service provision cannot account for any SA/CS changes. While day-care was closed, the number of inpatients for psychiatric care was not meaningfully decreased during the pandemic especially regarding acute and emergency care.

The first, and probably most striking finding, is that the number of SA significantly decreased by 31% between baseline and the pandemic (March–December 2019 and March–December 2020). There is almost no similar data from regions using a comparable design to Frappe, however, our findings are in line with the few previous reports on SA during the pandemic. The largest study to date [18] analyzed data from the French national hospital discharge database, which uses ICD-10 X-codes (X60 to X84) as also recorded in our study. In line with our study, a 16% reduction in SA was found during and after the lockdown period (defined as 16th of March until 7th of July), which is lower than the 31% we observed. However, in contrast to our data, this group found an increase in violent/severe SA and a decrease of SA by intoxication. The underlying reasons are unclear; changes in admission and referral patters which occurred differentially in different countries appear to be likely. As the French emergency and intensive care systems were affected harder by the pandemic in comparison to the German care system (as evident by the fact that French patients were flown out to Germany), it might have been the case that “milder” cases of SA were not admitted to hospitals in France at that time. The discrepant findings underscore the problems of hospital-based registries when it comes to the epidemiology of suicidal behaviors and call for unbiased, epidemiological databases that record SA and CS from both in- and outpatient services. Furthermore, a British study using primary care electronic records found that the incidence of self-harm decreased by approximately 38% following March 2020; interestingly, this was most pronounced in females and younger patients (< 45 years). In further support for a reduction in SA during the pandemic, data from Michigan [19] argue for a 40% reduction in the number of contacts to emergency departments for SA and intentional self-harm between March to December 2020. Collectively, our data, and those from the French, US and British studies, argue for a relevant decrease in SA from March 2020 to December 2020 as compared to the same period in 2019.

Several explanations for these apparently paradoxical findings are possible, ranging from psychological explanations of resource activation in the face of adversity (i.e. the “pulling-together effect”), to reduced stressor load especially in the acute, initial phase of the lockdown period, or due to reduced stress load regarding interpersonal conflicts during home–office periods. However, the fact that the rate of CS remained constant in the same region(s) and during the period argues against an actual reduction of SA. Thus, we propose that a larger number of SA remained undetected and that less patients were hospitalized after SA during the lockdown, which is supported by analyses of patient-related variables. Lockdown measures and COVID-19-related restrictions led to significantly less mobility in the whole of Germany. Accordingly, less people from outside Frankfurt/Main attempted suicide in the city during the pandemic as compared to the baseline period. Additionally, the sharp decrease in mobility may explain the reduction of SA in relation to public transport locations: while in the 2019 period, 61 SA occurred at the airport, train stations, at railways and in traffic, this number reduced to 11 in 2020. This reduction cannot reasonably be attributed to a reduction in the level of reporting, but more likely that a real reduction of SA in public areas occurred during the pandemic. This is, evidently, paralleled by a reduction in the SA methods “leaping from heights” and “railway/traffic”. Several studies found unchanged [20] or even increased [21] rates of traumatic SA; however, as the total number of SA were not registered in these studies their interpretation is difficult. Furthermore, lockdown measures as such might have an effect. The shutdown of restaurants, nightclubs, cultural life, etc., and the corresponding evacuation of inner-city spaces on weekends could underlie the striking reversal in the weekly pattern of SA in our dataset where the SA peak shifted from Sunday to Wednesday (Fig. 1a). This is also supported by the fact that SA after midnight decreased (Fig. 1b). The reduction of alcohol consumption in public spaces might have a role for both observations, and probably has contributed to the reduction in the number of SA. This is in line with pertinent recommendations to restrict drinking in public places as a means to reduce suicides [22].

Paralleling the reduced rate of SA in public areas, the rate of SA occurring at home increased significantly. Supporting the above arguments relating to alcohol in public spaces, both the rate (see Table 2) and absolute number of SA using intoxication as a method increased. Given that here is no convincing reason why the CS:SA ratio should change within such a short time frame, the most parsimonious explanation (apart from a reduction in public alcohol consumption and reduction of mobility) is that there was a reduction in the number of patients admitted to a psychiatric hospital after a SA during the pandemic. Given the pattern of SA-related factors, it seems conceivable that more patients attempted suicide at home, rather than in public spaces during the pandemic. This observation is in keeping with that fact that more patients used the method of intoxication (with lesser likelihood of hospitalization) in comparison to leaping from heights or attempted railway suicide. It is likely that such patients would not get in touch with medical emergency services at all, or might go unnoticed as having a SA. Furthermore, patients could have shied away from entering hospitals for the fear of SARS-CoV2 infection and warnings to reduce social contact. This is worrisome, as most of these patients suffer from a mental illness, and as SA is one of the most relevant risk factors for completed suicide [14]. Hence, a substantial number of patients suffering from mental illness might not have received adequate care during the pandemic, which will place them at risk for further suicidal acts/attempts. Measures to identify and approach this population are therefore urgently needed to prevent a rise in completed suicides in the aftermath of the COVID-19 pandemic.

### Limitations

Certainly, there are several limitations to our study. First and foremost, only SA seen in psychiatric hospitals and attached emergency rooms were documented, but not SA seen exclusively outside of hospitals or in one of the other six Frankfurt hospitals that provide emergency, but not psychiatric care (BG Unfallklinik, Bürgerhospital, Hospital zum Heiligen Geist, Krankenhaus Nordwest, St. Katharinen-Krankenhaus and St. Elisabethen-Krankenhaus), which might explain a share of the dark figure of around 50% given above. However, patients admitted after SA are referred to one of the institutions that participated in the present study. There is no reason to assume that referral patterns changed during the pandemic.

More uncertainty exists regarding the cases seen by practitioners only; however, even if reduced referral to psychiatric hospitals would fully account for the difference between baseline and the pandemic, this would not invalidate our conclusion that mental health care provision to those with greatest need was reduced in 2020. Our data should, therefore, draw attention to improve public mental health services to prevent an increase in suicidal behaviors in the aftermath of the COVID-19 pandemic. Further studies that monitor secular trends in suicidal behaviors from all healthcare sectors are urgently needed to allow preventive measures; the fact that no effective surveillance system for SA/CS is in place despite the high death toll is pointing to a huge unmet medical need that needs to be addressed.

## Supplementary Information

Below is the link to the electronic supplementary material.

Supplementary file1 (DOCX 14 kb)

Supplementary file2 (PDF 215 kb)

Supplementary file3 (PDF 267 kb)

Supplementary file4 (PDF 211 kb)
