# Supplementary material for: Changes in the pattern of suicides and suicide attempt admissions in relation to the COVID-19 pandemic
Source: Eur Arch Psychiatry Clin Neurosci. 2022 Jul 4;273(2):357–65. doi: 10.1007/s00406-022-01448-y (PMC9252546; doi:10.1007/s00406-022-01448-y)
Supplement: Supplementary file 1 — Supplementary file1 (DOCX 14 kb) [file 406_2022_1448_MOESM1_ESM.docx]

**Supplementary Table 1:** Household composition in patients presenting with SA at baseline and during the pandemic

|  | Suicide attempts | | | Completed suicides | | |
| --- | --- | --- | --- | --- | --- | --- |
| Percentage of patients living… | 03/19 – 12/19 | 03/20 – 12/20 | p-value overall | 03/19 – 12/19 | 03/20 – 12/20 | p-value (overall) |
| alone | 32.8 | 38.1 | 0.012 | 51.2 | 49.4 | 0.232 |
| without partner, with children | 2.5 | 5.0 |  | 4.7 | 1.2 |  |
| with partner, without children | 12.2 | 14.7 |  | 22.1 | 16.1 |  |
| with partner and children | 7.6 | 7.9 |  | 5.8 | 2.5 |  |
| with parents or relatives | 11.2 | 8.6 |  | 7.0 | 7.4 |  |
| with friends | 1.0 | 2.5 |  | 0.0 | 4.9 |  |
| homeless | 9.4 | 7.6 |  | 2.3 | 6.2 |  |
| imprisoned | 0.3 | 0.0 |  | 0.0 | 2.5 |  |
| in a psychiatric institution | 2.0 | 4.0 |  | 1.2 | 1.2 |  |
| in a nursing home | 1.3 | 0.7 |  | 4.7 | 4.9 |  |
| elsewhere | 11.7 | 4.0 |  | 0.0 | 1.2 |  |
| unknown | 7.9 | 6.8 |  | 1.2 | 2.5 |  |

**Supplementary Table 2:** Occupational status in patients presenting with SA at baseline and during the pandemic

|  | Suicide attempts | | | Completed suicides | | |
| --- | --- | --- | --- | --- | --- | --- |
| Percentage of patients … | 03/19 – 12/19 | 03/20 – 12/20 | p-value (overall) | 03/19 – 12/19 | 03/20 – 12/20 | p-value (overall) |
| working full time | 13.8 | 19.0 | <0.001 | 22.1 | 22.2 | 0.295 |
| working part time | 5.4 | 4.4 |  | 3.5 | 4.9 |  |
| retired | 15.1 | 18.2 |  | 36.0 | 29.6 |  |
| in education | 8.7 | 6.9 |  | 8.1 | 2.5 |  |
| unemployed | 19.0 | 25.9 |  | 16.3 | 12.3 |  |
| in sheltered workspaces | 1.3 | 3.3 |  | 0.0 | 0.0 |  |
| with other occupation | 17.4 | 6.6 |  | 3.5 | 4.9 |  |
| unknown | 19.2 | 15.7 |  | 10.5 | 28.4 |  |

**Supplementary Table 3.** Major underlying psychiatric diagnosis in patients attempting suicide

|  | Suicide attempts | | | Completed suicides | | |
| --- | --- | --- | --- | --- | --- | --- |
| Major diagnosis according  to ICD-10 category (%) | 03/19 – 12/19 | 03/20 – 12/20 | p-value (overall) | 03/19 – 12/19 | 03/20 – 12/20 | p-value (overall) |
| No ICD-10 diagnosis | 1.0 | 0.7 | 0.082 | 46.5 | 58.0 |  |
| F0x | 1.8 | 1.8 |  | 1.2 | 1.2 |  |
| F1x | 21.1 | 20.3 |  | 7.0 | 11.1 |  |
| F2x | 13.7 | 18.1 |  | 9.3 | 11.1 |  |
| F3x | 37.9 | 37.7 |  | 33.7 | 27.2 |  |
| F4x | 14.2 | 10.5 |  | 5.8 | 1.2 |  |
| F5x | 0.3 | 0.7 |  | 5.8 | 1.2 |  |
| F6x | 9.9 | 9.8 |  | 7.0 | 4.9 |  |
| F7x | 0.0 | 0.4 |  | 0.0 | 1.2 |  |
