# Supplementary material for: Changes in the pattern of suicides and suicide attempt admissions in relation to the COVID-19 pandemic
Source: Eur Arch Psychiatry Clin Neurosci. 2022 Jul 4;273(2):357–65. doi: 10.1007/s00406-022-01448-y (PMC9252546; doi:10.1007/s00406-022-01448-y)
Supplement: Supplementary file 2 — Supplementary file2 (PDF 215 kb) [file 406_2022_1448_MOESM2_ESM.pdf]

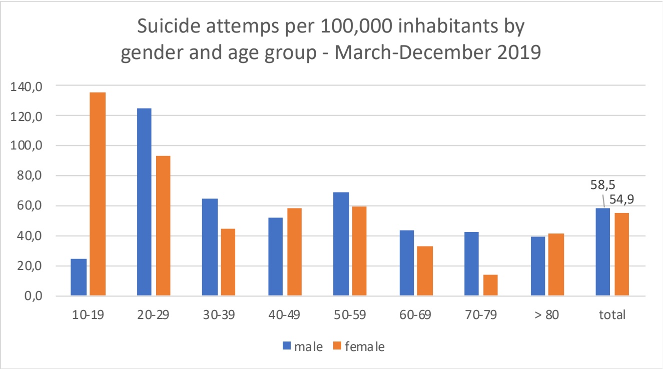
Suicide attemps per 100,000 inhabitants by gender and age group - March-December 2019
140,0
120,0
100,0
80,0
60,0
40,0
20,0
0,0
10-19
20-29
30-39
40-49
50-59
60-69
70-79
> 80
total
58,5
54,9
male
female

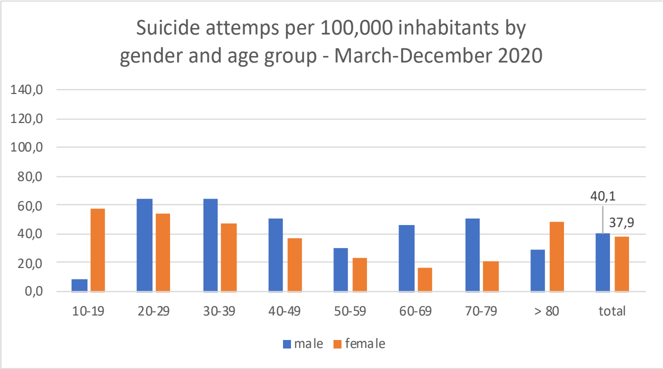
Suicide attemps per 100,000 inhabitants by gender and age group - March-December 2020
140,0
120,0
100,0
80,0
60,0
40,0
20,0
0,0
10-19
20-29
30-39
40-49
50-59
60-69
70-79
> 80
total
40,1
37,9
male
female

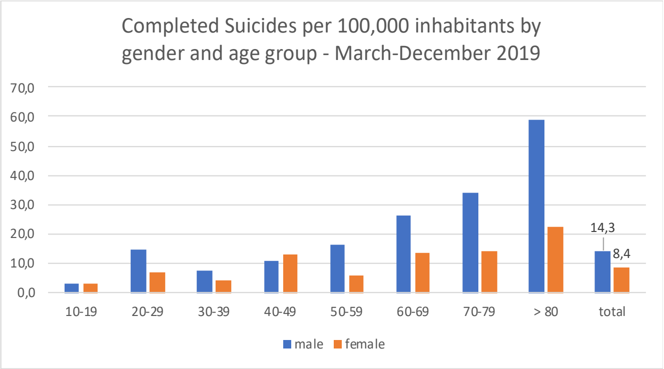
Completed Suicides per 100,000 inhabitants by gender and age group - March-December 2019
70,0
60,0
50,0
40,0
30,0
20,0
10,0
0,0
10-19
20-29
30-39
40-49
50-59
60-69
70-79
> 80
total
14,3
8,4
male
female

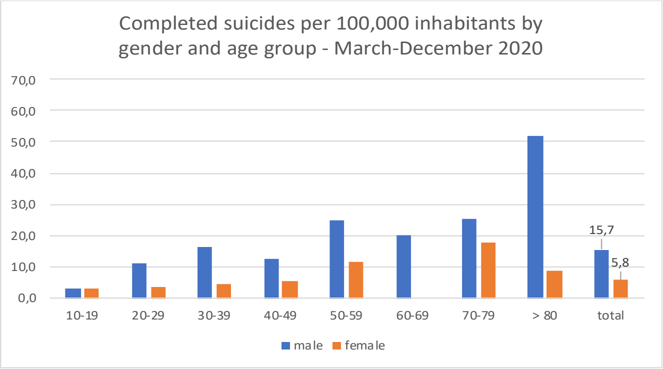
Completed suicides per 100,000 inhabitants by gender and age group - March-December 2020
70,0
60,0
50,0
40,0
30,0
20,0
10,0
0,0
10-19
20-29
30-39
40-49
50-59
60-69
70-79
> 80
total
15,7
5,8
male
female
