# Supplementary figures and images for: Changes in the pattern of suicides and suicide attempt admissions in relation to the COVID-19 pandemic
Source: Eur Arch Psychiatry Clin Neurosci. 2022 Jul 4;273(2):357–65. doi: 10.1007/s00406-022-01448-y (PMC9252546; doi:10.1007/s00406-022-01448-y)

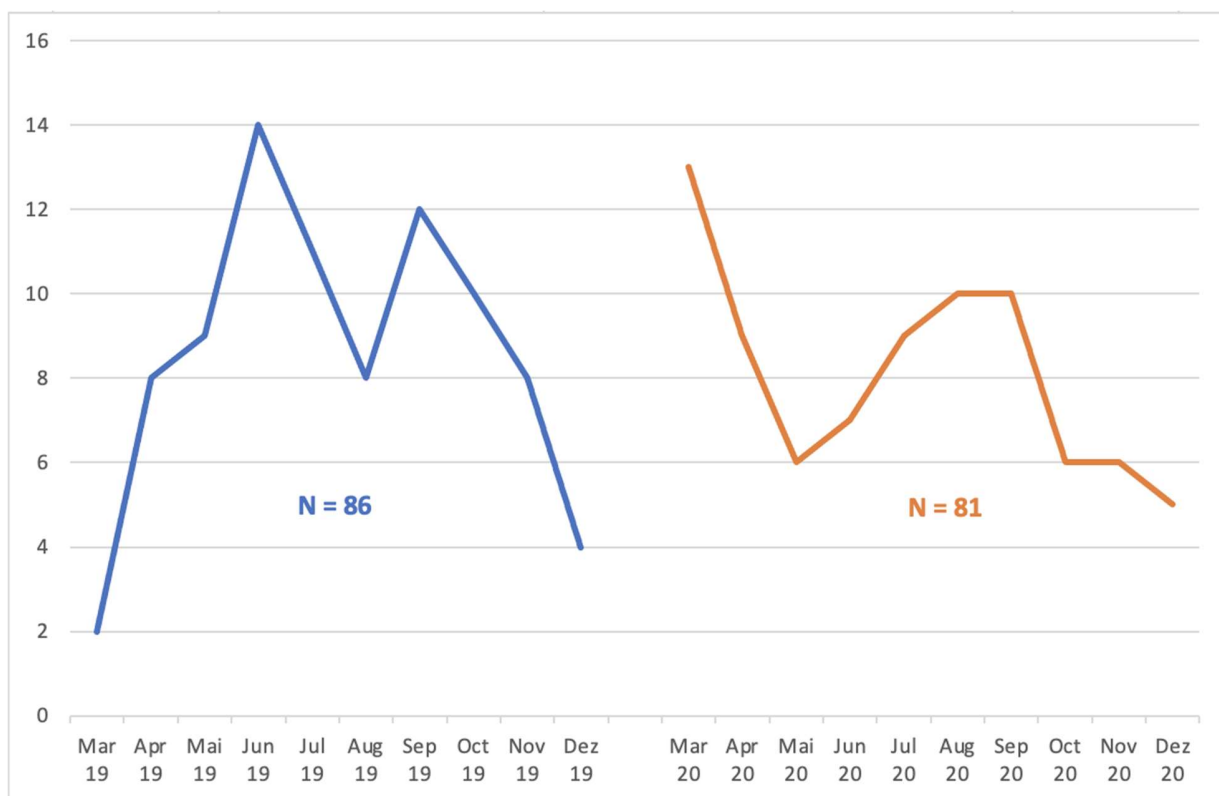

Supplement: Supplementary file 3 — Supplementary file3 (PDF 267 kb) [file 406_2022_1448_MOESM3_ESM.pdf]

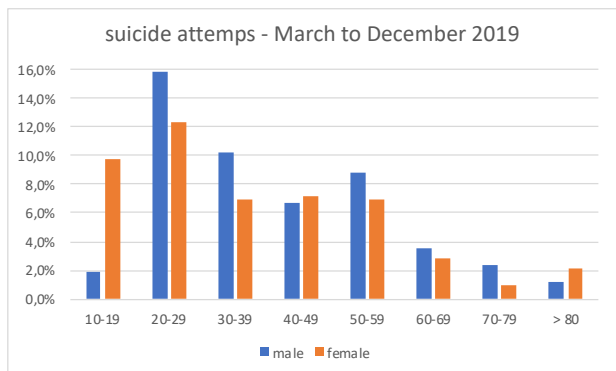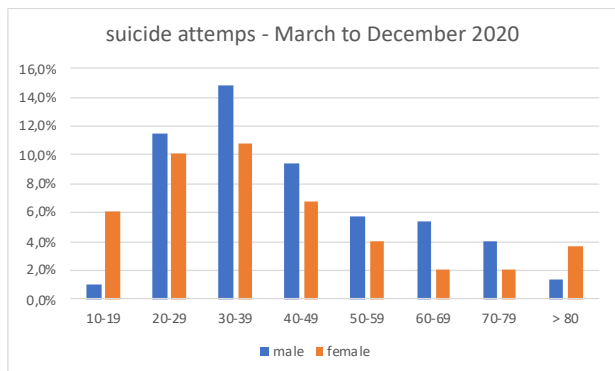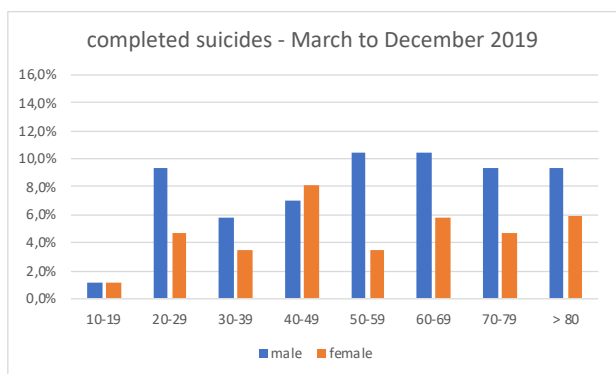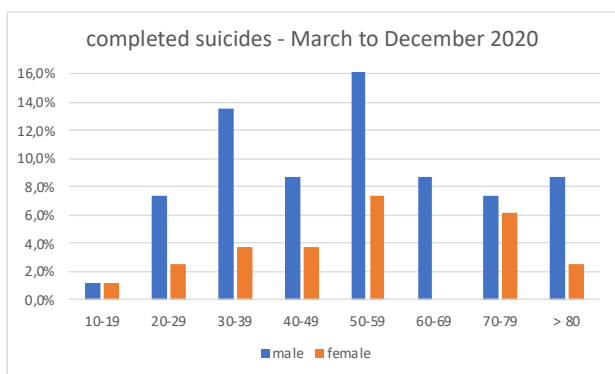

Supplement: Supplementary file 4 — Supplementary file4 (PDF 211 kb) [file 406_2022_1448_MOESM4_ESM.pdf]
